# Ultrasound triggered microbubbles enhance the diagnosis and therapy of colorectal cancer

**DOI:** 10.1186/s11671-026-04872-4

**Published:** 2026-09-18

**Authors:** Qingyun Wang, Yun Gong, Shengguo Zao, Feng Sun, Jiaping Wang, Yongping Lu

**Affiliations:** 1 Department of Gastrointestinal Surgery II, The Second Affiliated Hospital of Kunming Medical University, Kunming, 650000 China; 2 Radiology Department, The Second Affiliated Hospital of Kunming Medical University, Kunming, 650000 Yunnan China; 3 Ultrasound Department, Yunnan University Affiliated Hospital, Kunming, 650021 Yunnan China

**Keywords:** Ultrasound-triggered microbubbles, Colorectal cancer, Diagnosis, Therapy, Action mechanism, Tumor microenvironment

## Abstract

As an emerging integrated diagnostic and therapeutic platform, ultrasound-triggered microbubbles (UTMBs) have demonstrated significant potential in the treatment of colorectal cancer (CRC). This article introduces the different types of UTMBs, including micron-sized bubbles, nanobubbles, and nanodroplets) and describes their respective structural characteristics. These characteristics directly influence the behavior and function of UTMBs in vivo. The mechanisms underlying UTMB-mediated tumor therapy are systematically summarized, including physical effects (such as cavitation, acoustic streaming, and thermal effects), modulation of the tumor microenvironment (TME), and synergistic interactions with therapeutic agents. In addition, the diagnostic applications of UTMBs in CRC are discussed, including contrast-enhanced ultrasound (CEUS) imaging, molecular marker detection, and monitoring of treatment response. The potential applications of UTMBs in chemotherapy sensitization, immunotherapy synergy, and gene therapy are further highlighted. Finally, the challenges and limitations of UTMBs in CRC diagnosis and treatment are analyzed, and future development directions are outlined. This review aims to provide a comprehensive reference and guidance for further research and clinical translation.

## Introduction

CRC remains one of the most common and deadliest cancers worldwide, posing a significant threat to human health. Although substantial progress has been made in early screening, diagnosis, and treatment, the prognosis of patients with advanced CRC remains poor. This is mainly attributed to tumor heterogeneity, high propensity for metastasis, resistance to conventional therapies (such as chemotherapy and radiotherapy), and the complex TME, which limits therapeutic efficacy [1–3]. Traditional diagnostic methods, such as colonoscopy and CT/MRI imaging, play an important role in disease detection and staging; however, they also have limitations, including procedural discomfort, potential radiation exposure, and limited sensitivity for early lesions and micrometastases [4, 5]. Systemic chemotherapy is often associated with significant toxicity, and achieving sufficient drug accumulation at the tumor site remains challenging. Emerging targeted therapies and immunotherapies also face challenges due to the development of drug resistance and TME-mediated immunosuppression [6, 7]. Therefore, there is an urgent need for integrated diagnostic and therapeutic strategies. Such strategies should provide high accuracy, low toxicity, real-time monitoring capabilities, and improved therapeutic outcomes for patients with CRC.

UTMBs are a novel therapeutic technology with considerable potential for cancer treatment, particularly in CRC. Microbubbles are gas-filled microspheres composed of an inert gas core and an outer shell made of biocompatible materials (such as lipids, polymers, or albumin). They are typically 1–10 μm in diameter, a size comparable to that of red blood cells, allowing them to circulate within the vasculature and function as ultrasound contrast agents [8–10]. When exposed to ultrasound, these microbubbles elicit a range of physical and biological effects, enabling imaging, targeted drug delivery, gene transfection, and modulation of the TME [11–13]. Compared with traditional methods, UTMB technology offers several advantages, including non-invasiveness, real-time imaging, the absence of ionizing radiation, good biocompatibility, and potential for repeated use, making it a promising approach to overcoming current limitations in CRC diagnosis and treatment.

This review aims to summarize recent advances in the use of UTMBs for the diagnosis and treatment of CRC. We discuss in detail the different types of UTMBs and their mechanisms of action in tumor therapy. It also examines their applications in CRC diagnosis, including enhanced tumor imaging, molecular imaging, and monitoring of treatment response. In addition, UTMBs are highlighted for their therapeutic potential in CRC, such as enhancing chemotherapy efficacy, facilitating immunotherapy, and enabling gene therapy. Finally, this review addresses current challenges, controversies, and limitations in the field, and outlines future research directions and potential clinical applications. We hope that this comprehensive analysis will provide a theoretical foundation and practical guidance for advancing the clinical application of UTMBs in CRC management.

## Types of ultrasound-triggered microbubbles, nanobubbles, and nanodroplets

The design and preparation of UTMBs constitute the foundation for their application in the diagnosis and treatment of CRC. These microbubbles typically consist of a gas core and a biocompatible shell. Their physicochemical properties, including size, shell material, gas type, and surface modification, directly affect their in vivo stability, pharmacokinetics, targeting capability, and ultrasound response characteristics [11, 14].

### Structural composition and size classification of microbubbles

The gas core of UTMBs typically consists of inert gases such as sulfur hexafluoride (SF6), perfluoropropane (C3F8), and perfluorobutane (PFB), whose low aqueous solubility and slow diffusion contribute to prolonged circulation and enhanced ultrasound contrast [15, 16]. Phase-change perfluorocarbons such as perfluoropentane (PFP) can undergo acoustic droplet vaporization under ultrasound stimulation, enabling localized drug release and enhanced imaging [16, 17]. The shell composition further dictates the stability, circulation behaviour, and acoustic responsiveness of UTMBs. Lipid-based microbubbles exhibit excellent biocompatibility, whereas polymer-based microbubbles offer greater mechanical stability and longer circulation times [18, 19]. Based on particle size, UTMBs are broadly classified into microbubbles (MBs), nanobubbles (NBs), and nanodroplets (NDs). MBs primarily function as intravascular contrast agents and enhance vascular permeability through cavitation, whereas NBs and NDs exhibit improved tumour penetration, facilitating deeper drug delivery, molecular imaging, and theranostic applications [8, 13, 20–24] (Table 1).

**Table 1:** Comparison of different types of ultrasound-triggered microbubbles, nanobubbles, and nanodroplets

Types	Size range	Key features	Primary applications	Advantages	Limitations	Representative literature
Micrometer-scale microbubbles (MBs)	1–10 µm	Intravascular contrast agent, cavitation effect	Enhanced imaging, increased vascular permeability	Strong ultrasound signal, easy to prepare	Cannot penetrate vessel walls, relatively short circulation time	[8, 20]
Nanoscale bubbles (NBs)	< 1 µm	High tissue permeability	Tumor interstitial drug delivery, molecular imaging	Penetrates vascular walls, EPR effect	Lower stability than MBs, relatively weak ultrasound signal	[21, 25]
Nanodroplets (NDs)	< 1 µm (droplet)	Acoustic Droplet Vaporization (ADV)	Precise drug release, imaging enhancement	Combines nano-carrier permeability advantages with microbubble ultrasonic responsiveness	ADV triggering conditions require precise control and complex preparation	[13, 17]

### Targeted strategies and drug delivery

To enhance tumour-specific accumulation, UTMBs employ both passive and active targeting strategies. Passive targeting is primarily mediated by the enhanced permeability and retention (EPR) effect, which allows nanoscale carriers to preferentially accumulate in tumour tissues owing to abnormal vascular permeability and impaired lymphatic drainage [25, 26]. Although the EPR effect remains heterogeneous across human tumours, clinical evidence supports its relevance in colorectal cancer (CRC) and underscores the need for individualized optimization of UTMB-based strategies [27–29] Active targeting is achieved through the conjugation of specific ligands to UTMBs, enabling recognition of molecular markers overexpressed on tumour cells or tumour-associated vascular endothelial cells. Representative targets include angiogenesis-related markers such as VEGFR2 [30, 31], tumour invasion-associated molecules such as CXCR4 [32], and immune-related targets including B7-H3, CD93, and PD-L1 [33–36]. Folate receptor-targeting has also been explored to further improve tumour selectivity. Collectively, these strategies provide an important foundation for molecular imaging and targeted therapy in CRC.

**Fig. 1:**
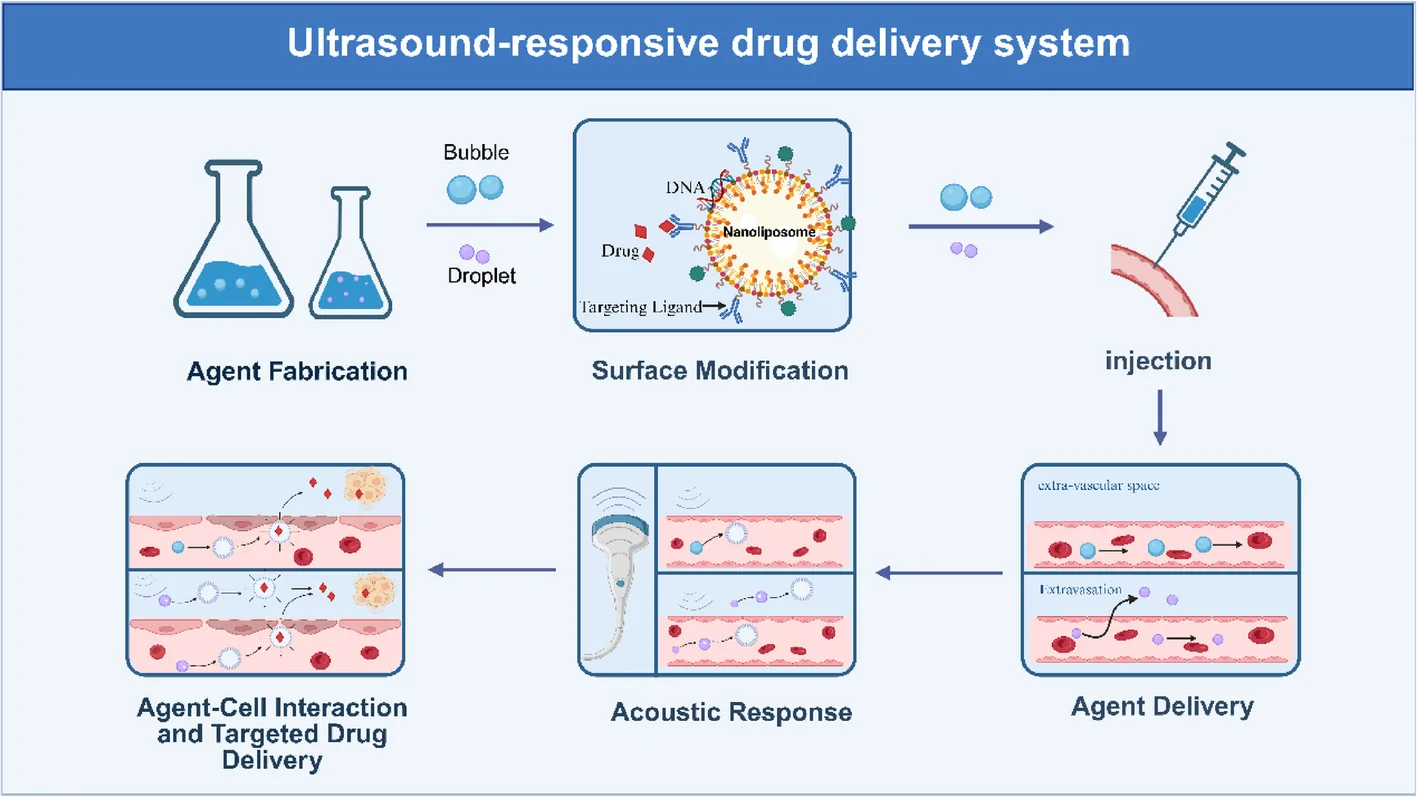
Schematic illustration of ultrasound-targeted microbubble (UTMB)-mediated drug delivery. UTMBs are engineered through surface functionalization and therapeutic payload encapsulation to achieve targeted delivery. Following intravenous administration, microbubbles and nanodroplets accumulate within tumor-associated vasculature and extravasate into the tumor microenvironment. Upon ultrasound stimulation, acoustic cavitation and sonoporation effects enhance vascular permeability and transient membrane permeabilization, thereby facilitating localized drug release and intracellular delivery of therapeutic agents. Created with BioRender.com

To further improve delivery precision, UTMBs can also function as carriers for chemotherapeutic agents, gene vectors, and immunomodulatory molecules. Therapeutic payloads can be encapsulated within the shell or core of UTMBs [37, 38] or incorporated into nanoparticles attached to their surface, thereby combining the loading capacity of nanocarriers with ultrasound-responsive release. This design further enhances spatiotemporal control over drug delivery and expands the therapeutic potential of UTMB-based platforms [39, 40] (Fig. 1).

## Action mechanisms of ultrasound-triggered microbubbles in tumor therapy

This section focuses on general mechanisms, with examples from different cancers. The roles of UTMBs in tumor treatment are diverse and primarily involve the physical effects produced by ultrasound and microbubbles, biological modulation of the TME, and synergy with therapeutic drugs. Collectively, these mechanisms help deliver therapeutic agents, induce tumor cell death, and enhance the immune response.

### Physical effects

The interaction between ultrasound and microbubbles underpins the therapeutic effects of UTMBs, primarily through cavitation, acoustic streaming, and thermal effects [8, 12, 13].

#### Cavitation effect

Cavitation refers to the nucleation, growth, and collapse of microscopic gas cavities in a liquid under the influence of ultrasonic waves. Based on ultrasonic intensity and microbubble behavior, cavitation can be classified into stable cavitation and inertial cavitation [8, 41, 42].

In Stable cavitation, under low Mechanical Index (MI) ultrasound, microbubbles undergo periodic expansion and contraction within the acoustic field without rupturing. This stable oscillation generates microscopic shear forces, microflows, and localized pressure changes, thereby increasing vascular endothelial cell permeability and promoting drug leakage from blood vessels into the tumor stroma [8, 20, 43]. Tang et al. [43] demonstrated that diagnostic ultrasound and microbubble-induced sononeoperfusion can enhance tumor blood perfusion and improve the delivery efficiency of doxorubicin in Walker-256 rat tumors. In contrast, Inertial cavitation occurs under the action of high mechanical index ultrasound, where microbubbles expand violently and then collapse rapidly, producing local high temperature, high pressure, and shock waves. This mechanical disruption can induce transient pore formation in tumour cell membranes (sonoporation), leading to direct cellular damage and facilitating the intracellular delivery of macromolecular drugs and genes [8, 20, 44–49]. Roy et al. [47] found, in a 3D spherical model, that microbubble-assisted ultrasound enabled spheroids to form channels and enhanced bleomycin’s cytotoxicity. In addition, inertial cavitation can also damage tumor blood vessels, causing ischemia and reperfusion injury, thereby amplifying the anti-tumor effect of chemotherapeutic drugs [41, 50]. Wu et al. [50] found that locally inducing tumor ischemia and reperfusion by UTMD can significantly enhance the anti-tumor efficacy of doxorubicin chemotherapy. Real-time monitoring and control of cavitation activity are essential for optimizing therapeutic efficacy and ensuring safety. Pakdaman Zangabad et al*.* [51] developed a highly sensitive passive cavitation detector based on a capacitive micromachined ultrasound transducer (CMUT) for tracking the dynamic behavior of microbubbles during focused ultrasound treatment. However, Wu et al. [52, 53] also noted that the simple dichotomy of cavitation types based on acoustic signals poses challenges and requires more refined monitoring standards.

#### Sound stream effect

The acoustic flow effect is the phenomenon in which, under the influence of ultrasonic waves, a directional flow occurs in a liquid. When microbubbles vibrate in the ultrasonic field, they induce the surrounding tiny liquid to move with them. This movement is known as microstreaming [54–56]. This microstreaming generates shear stress, altering the permeability of cell membranes and enhancing the drug’s ability to spread and penetrate tumor tissue. Research by Memari et al. [55] demonstrated that shear stress induced by focused ultrasound via microbubbles can regulate the immunobiological characteristics of endothelial cells, affect the expression of cell adhesion molecules, and influence cytokine secretion. This mechanism may enhance the efficacy of cellular immunotherapy for solid tumors.

#### Thermal effect

High-Intensity Focused Ultrasound (HIFU) can directly ablate tumor tissue via thermal effects, with microbubbles significantly enhancing these effects [16, 57]. The oscillation and rupture of microbubbles under ultrasonic irradiation generate localized heat, thereby lowering the HIFU ablation threshold and improving treatment efficiency. Chen et al. [16] demonstrated that dual-frequency HIFU, combined with perfluoropentane-loaded polymer nanoparticles, significantly reduced the acoustic intensity threshold for thermal damage while improving tumor suppression rates. However, in drug delivery applications, lower ultrasound frequencies are typically used to avoid excessive thermal damage, with a primary focus on leveraging cavitation and acoustic streaming.

### Biological regulation of the tumor microenvironment

UTMB not only facilitates drug delivery through physical effects but also significantly modulates the TME, thereby strengthening the anti-tumor immune response and overcoming therapeutic drug resistance.

#### Increased vascular permeability and drug extravasation

The interaction between ultrasound and microbubbles can temporarily disrupt tight junctions between tumor vascular endothelial cells, thereby increasing vascular permeability and promoting the extravasation of macromolecular drugs, nanocarriers, and immune cells from blood vessels into the tumor stroma [8, 20, 58–61]. Ingram et al. [58] demonstrated that ultrasound-triggered VEGFR2-targeted microbubbles significantly increased tumor drug accumulation by up to 2.5-fold, while simultaneously reducing drug bioavailability in normal tissues, thereby enhancing the therapeutic index of chemotherapeutic agents. This enhanced vascular permeability is one of the key mechanisms by which UTMBs potentiate the effects of chemotherapy, gene therapy, and immunotherapy. Moreover, fluid flow itself plays a critical role. Memari and Helfield observed that under identical ultrasound conditions, microbubbles with faster flow increased endothelial cell membrane permeability by at least 2.3-fold compared to slower flow. Therefore, local fluid flow conditions significantly impact the efficacy of UTMBs.

#### Immune system regulation

The immunoregulatory role of UTMBs in TME has become a prominent area of research in recent years, particularly in the context of synergistic immune checkpoint inhibitor (ICI) therapy [62, 63].

Promotion of immune cell infiltration: UTMD has been shown to enhance the infiltration of CD8+ T cells, dendritic cells (DCs), and M1 macrophages within tumors, while reducing the presence of immunosuppressive cells such as regulatory T cells (Tregs) and myeloid-derived suppressor cells (MDSCs), thereby reversing the immunosuppressive TME [59, 60, 64–68]. For example, Jahangiri et al. [64] observed that ultrasound-triggered microbubble cavitation (UTMC), combined with CD39 knockout, increased extracellular ATP levels, vascular inflammation, and cancer cell death. This approach synergized with anti-PDL1 antibodies to reduce tumor growth and promote the infiltration of increased cytotoxic T lymphocytes (CTLs), DCs, and M1.

Induction of Immunogenic Cell Death (ICD): UTMD can drive tumor cells to undergo ICD, leading to the release of intracellular danger-associated molecular patterns (DAMPs), such as ATP and calreticulin (CRT). These molecules activate dendritic cells, initiating anti-tumor immune responses [67, 69, 70]. Zhao et al. [70] developed a nanoscale ultrasound contrast agent that triggers nitric oxide (NO) release via UTMD, thereby synergistically enhancing immunotherapy efficacy when combining with paclitaxel-induced ICD.

Remodeling the Immunosuppressive Microenvironment: UTMDs can facilitate the polarization of M2 macrophages toward M1 macrophages, alleviate tumor hypoxia, and modulate the functions of other immunosuppressive cells, thereby enhancing anti-tumor immunity [59–61, 66, 68, 71, 72]. Zheng et al. [66] demonstrated that a drug-loaded microvesicle delivery system combined with αPD-L1 can remodel the TME, promote M2-to-M1 macrophage polarization, reduce MDSCs, and thereby enhance the efficacy of PD-L1 blockade immunotherapy.

#### Reduce tumor hypoxia

Tumor tissues are often hypoxic, which facilitates tumor progression, angiogenesis, and resistance to radiotherapy and chemotherapy [3]. UTMBs can alleviate tumor hypoxia by enhancing tumor vascular perfusion and improving oxygen delivery within the tumor, thereby increasing treatment sensitivity [70, 71, 73]. Huang et al. [73] demonstrated that ultrasound-controlled drug release prevents pro-tumorigenic transformation and improves treatment efficacy against residual hepatocellular carcinoma by maintaining the inflammatory microenvironment.

#### Extracellular matrix (ECM) remodeling

The extracellular matrix (ECM) of tumors acts as a physical barrier to drug penetration. Mechanical forces generated by UTMDs can degrade ECM components, thereby improving drug penetration and distribution within tumor tissues [74]. Ai et al. [74] developed cancer-associated fibroblast (CAF)-targeted nanodroplets combined with UTMDs to inhibit melanoma growth by enhancing drug release and ECM degradation.

### Mechanism of synergistic action with therapeutic agents

UTMBs can synergize with chemotherapeutic agents, immunotherapies, gene therapies, and other modalities to enhance anti-tumor effects. These mechanisms are discussed in detail in Sect. 3.2 (Therapy), in the context of CRC, where CRC-specific examples are provided.

Collectively, these mechanisms contribute to enhanced drug delivery, improved therapeutic efficacy, and modulation of the tumor microenvironment, thereby providing the mechanistic foundation for subsequent therapeutic applications of UTMBs in CRC.

## Diagnostic and therapeutic applications of ultrasound-triggered microbubbles in colorectal cancer

**Fig. 2:**
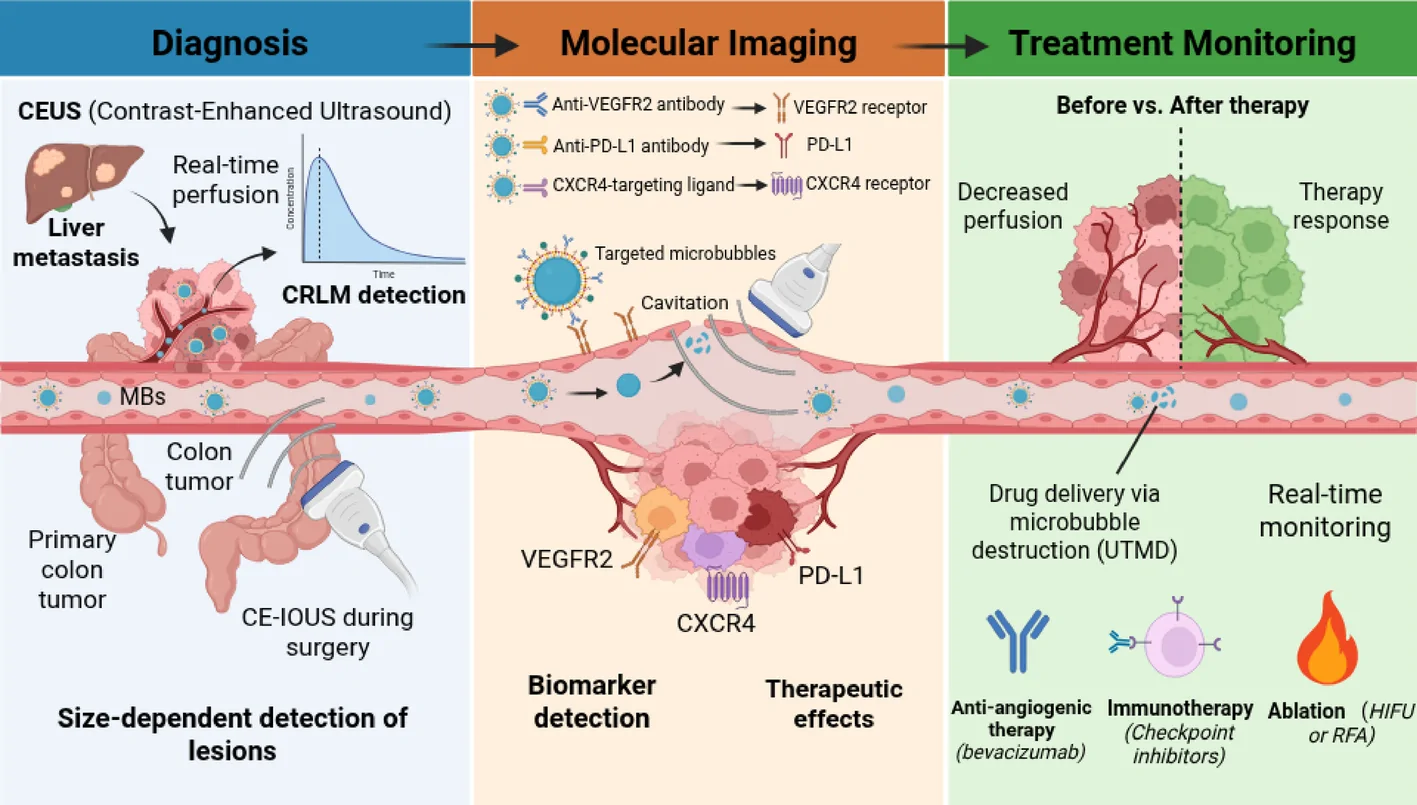
Diagnostic and imaging applications of UTMBs in colorectal cancer

UTMBs exhibit distinct advantages in diagnosing colon cancer (CRC), particularly in real-time imaging, molecular marker identification, and monitoring treatment efficacy. They possess non-invasive, high-resolution, and non-radiative properties, positioning UTMBs as a significant complement to conventional imaging techniques. Furthermore, UTMBs demonstrate considerable potential for therapeutic management of CRC, enhancing the efficacy of chemotherapy, immunotherapy, and gene therapy through various mechanisms. This offers promising avenues for addressing tumor drug resistance (Fig. 2).

### Diagnosis

Schematic overview of the diagnostic and imaging applications of ultrasound-targeted microbubble (UTMB) systems in colorectal cancer (CRC). Contrast-enhanced ultrasound (CEUS) enables real-time visualization of tumor perfusion and improves the detection of primary colorectal tumors and colorectal liver metastases (CRLM). Molecular imaging with targeted microbubbles facilitates the identification of tumor-associated biomarkers, including VEGFR2, PD-L1, and CXCR4, thereby supporting tumor characterization and therapeutic guidance. UTMB-based imaging also enables dynamic monitoring of treatment responses, including changes in tumor perfusion, therapeutic efficacy, and microenvironmental alterations during anti-angiogenic therapy, immunotherapy, and ablation therapy.

#### Contrast-enhanced ultrasound (CEUS)

CEUS utilizes microbubbles as a nanoscale intravascular contrast agent, enabling real-time, dynamic assessment of tissue perfusion and vascular structures, thereby enhancing tumor detection rates and characterization capabilities [4, 9]. Finding primary and metastatic CRC lesions. CEUS is particularly valuable for detecting primary CRC lesions and their metastases, especially colorectal cancer liver metastases (CRLM). The microbubbles remain within the tumor blood vessels, making tumor tissue appear brighter on ultrasound and allowing it to be easily distinguished from surrounding normal tissue. Qin et al. [15] conducted a study comparing the effect of microbubbles containing sulfur hexafluoride (SHF) and perfluorobutane (PFB) in discovering CRLM. The study found that SHF-CEUS and PFB-CEUS exhibit similar accuracy in detecting and characterizing lesions. However, SHF-CEUS demonstrated superior efficacy in identifying CRLMs larger than 10 mm, while PFB-CEUS was more effective in detecting smaller CRLMs (< 10 mm). These findings suggest that selecting different microbubble types based on lesion size may optimize diagnostic accuracy. Studies by Li et al. [75] and Shah et al. [76] further emphasized that for CRLM patients with non-resectable tumors, intraoperative contrast-enhanced ultrasound (CE-IUS) can improve post-surgical recurrence-free survival and assist in detecting liver metastases during surgery. CE-IUS altered the treatment plan for 39.9% of patients and significantly improved their prognosis. In addition to lesion detection, CEUS enables the assessment of tumor angiogenesis and perfusion. Tumor angiogenesis and perfusion assessment. Tumor angiogenesis is key to its growth and spread. CEUS allows for real-time visualization of blood flow within a tumor, providing detailed insights into vascularization, such as blood flow speed and volume [30, 77, 78]. Freeling and Rezvani [77] employed a miniature ultrasound device combining 3D reconstruction and specialized imaging techniques to study colon cancer in mice. This method can quantify tumor volume and blood vessel conditions, offering a more comprehensive view than conventional planar ultrasound. It allows for continuous monitoring of tumor size, shape, and vascular changes without causing harm to live animals. Zhou et al. [78] utilized three-dimensional dynamic contrast-enhanced ultrasound (3D DCE-US) to predict the therapeutic effect of bevacizumab, an anti-angiogenesis drug, in a mouse colon cancer model. Their results indicated that tumor perfusion parameters, such as peak enhancement and area under the curve, decreased significantly following treatment, demonstrating that these early changes can reliably predict the effectiveness of the therapy.

#### Molecular imaging for biomarker identification

Molecular imaging uses targeted microbubble-activated microbubbles to identify and bind to molecular markers that are specifically overexpressed on the surfaces of tumor cells or tumor vascular endothelial cells, thereby enabling early detection, staging, and molecular characterization of tumors [5, 9]. Although several targeted ultrasound imaging studies were initially developed in non-CRC tumor models, these strategies provide important technical references for the future application of molecular ultrasound imaging in CRC, particularly in the context of evaluating tumor angiogenesis and assessing the immune microenvironment.

**Fig. 3:**
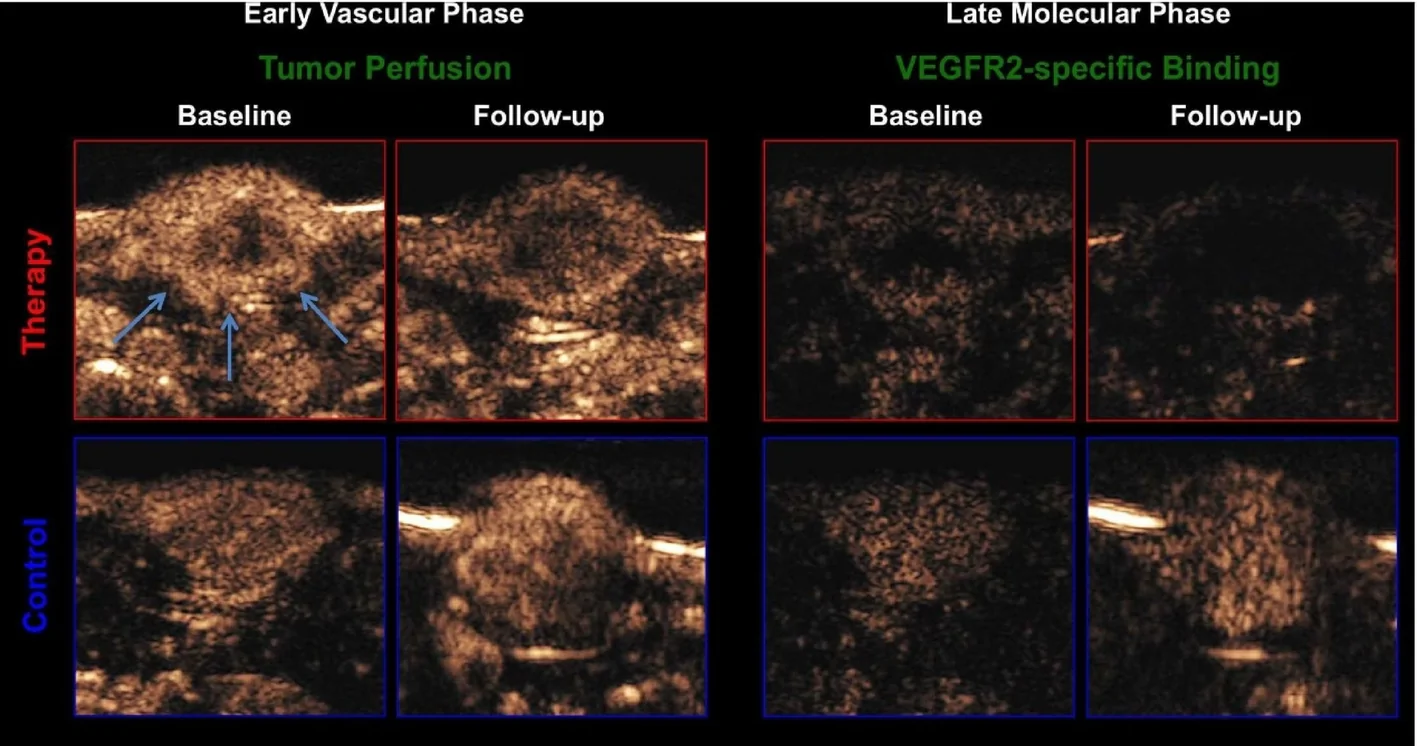
Representative CEUS images of rat subcutaneous colorectal adenocarcinoma xenografts using VEGFR2-targeted microbubbles (BR55) at baseline and 7-day follow-up. Left panels: early vascular phase (wash-in area under the curve, WiAUC) reflecting tumor perfusion. Right panels: late molecular phase (8 min post-injection) reflecting VEGFR2-specific binding. The regorafenib-treated group showed markedly reduced signal intensity in both phases at follow-up compared with baseline and with the control group, indicating decreased tumor perfusion and reduced VEGFR2 expression. Reprinted from [31] under a CC BY license, with permission from the authors, original copyright 2017

Targeting tumor vascular markers: Tumor neovascular endothelial cells often express specific molecules, such as vascular endothelial growth factor receptor 2 (VEGFR2). Wang et al. [30] demonstrated that 3D molecular-targeted ultrasound imaging of VEGFR2 can accurately quantify its expression in colon cancer, providing better early anti-angiogenesis therapy. Eschbach et al. [31] similarly applied VEGFR2-targeted microbubble contrast ultrasound to monitor the therapeutic efficacy of regorafenib, a multi-target kinase inhibitor, in experimental colon adenocarcinoma, with validation against DCE-MRI and immunohistochemical. Likewise, Herr et al. [79] used VEGFR2-targeted microbubble ultrasound imaging to assess the efficacy of anti-PD-L1/anti-CTLA-4 combination immunotherapy in a melanoma model and observed significant reductions in both vessel density and VEGFR2 expression in the treated group. Huang et al. [80] further demonstrated that modulation-based acoustic radiation force (mARF) combined with VEGFR-2 targeted microbubbles enables effective detection of abdominal aortic aneurysms (AAAs) that correlate with VEGFR-2 expression levels. Targeting tumor cell or immune cell markers: In addition to vascular markers, microbubbles can target specific markers expressed on tumor cells or immune cells within the TME. For example, CXCR4 is highly overexpressed in various tumors. Qiu et al. [32] developed CXCR4-targeted microbubbles for molecular imaging of liver tumors and enhancement of chemotherapy and immunotherapy efficacy. B7-H3, an immune checkpoint molecule, is also highly expressed in multiple tumor types. Bam et al. [81] developed and evaluated B7-H3-targeted microbubbles, demonstrating their effectiveness in vivo ultrasound molecular imaging of breast cancer. Natarajan et al. [36] showed that ultrasonic molecular imaging (USMI) using targeted microbubbles (TMBs) can assess PD-L1 expression in tumor endothelial cells, thereby predicting patient response to ICIs. Wang et al. [34] utilized MMRN2-modified microvesicles targeting CD93 for ultrasound imaging and found that CD93 expression correlates with the immunosuppressed tumor microenvironment and reduced cytotoxic T-cells infiltration, suggesting its potential for evaluating tumor immune status. Thy1, highly expressed in pancreatic ductal adenocarcinoma (PDAC), has also been targeted. Bam et al. [35] developed a Thy1-targeted ultrasound contrast agent for early detection, which is expected to be used for the early detection of PDAC. Theranostics Applications: Molecularly triggered microbubbles serve not only diagnostic purposes but also enable targeted drug delivery, facilitating integrated diagnostic and therapy (Theranostics) [11, 26, 82]. For example, Abdipour et al. [19] synthesized multifunctional polymeric microbubbles loaded with gold nanoparticles for combined cancer imaging and therapy. Sahoo et al. [23] developed ultrasound-responsive nanodroplets that convert into microbubbles and generate reactive oxygen species. Zhu et al. [83] reported ultrasound-guided photodynamic therapy using magnetic black phosphorus microbubbles. Yang et al. [84] achieved precise tumor diagnosis and treatment using gossypol-crosslinked ultrafine iron oxide nanoclusters, combined with ultrasound-enhanced T1-weighted magnetic resonance imaging (T1-MR) and chemodynamic therapy. Ai et al. [74] developed CAF-targeted ultrasound-responsive nanodroplets that not only inhibit melanoma growth but also serve as contrast agents for tumor imaging (Fig. 3).

#### Monitoring treatment response

UTMBs provide real-time, dynamic, and quantitative advantages for monitoring treatment responses in CRC, enabling earlier and more accurate evaluation of therapeutic efficacy than traditional methods.

Evaluation of anti-angiogenic therapy efficacy: Anti-angiogenic drugs inhibit tumor angiogenesis. CEUS enables real-time assessment of tumor vascular perfusion, facilitating early evaluation of therapeutic response. Zhou et al. [78] demonstrated that three-dimensional dynamic contrast-enhanced ultrasound (3D DCE-US) revealed significant reductions in tumor perfusion parameters within one day following bevacizumab treatment and accurately predicted therapeutic outcomes. Wang et al. [30] further reported that molecularly targeted ultrasound combined with dynamic CEUS provides precise molecular and functional information on tumor neovascularization, thereby improving imaging accuracy.

Monitoring chemotherapy and immunotherapy efficacy: UTMBs enable visualization of drug accumulation and distribution within tumors, as well as dynamic changes in the TME induced by immunotherapy. Bellary et al. [85] utilized quantitative CEUS (qCEUS) to monitor gene therapy-induced blood flow alterations in neuroblastoma models, thereby optimizing the timing of chemotherapy administration. Huang et al. [73] demonstrated that ultrasound-mediated controlled drug release enhances therapeutic efficacy against residual liver tumors. Natarajan et al. [36] proposed that the combination of ultrasound molecular imaging (USMI) and triggered microbubbles may serve as a non-invasive biomarker for predicting responses to ICIs.

Evaluation of tumor ablation efficacy: During tumor ablation therapies, such as HIFU and radiofrequency ablation, CEUS enables real-time assessment of blood flow within the ablation zone, allowing determination of ablation completeness and reducing recurrence risk [86] reported in a clinical trial that UTMD combined with transarterial radioembolization (TARE) significantly improved tumor response in patients with hepatocellular carcinoma, without major safety concerns.

**Fig. 4:**
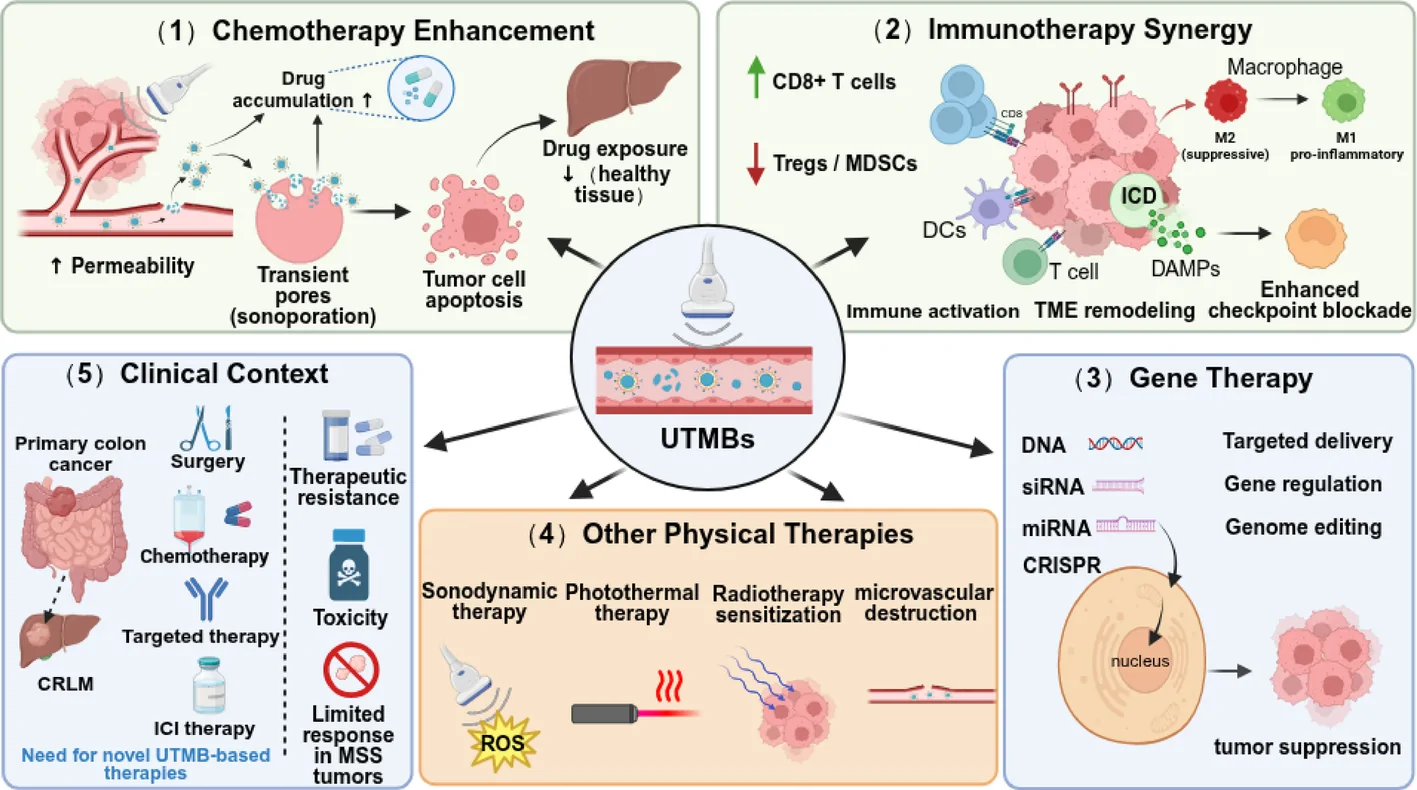
Therapeutic applications of UTMBs in colorectal cancer

Overall, current evidence suggests that UTMB-based imaging provides important advantages in CRC management by enabling real-time visualization of tumor perfusion, vascular heterogeneity, and molecular biomarker expression. Compared with conventional imaging modalities, targeted CEUS and molecular ultrasound imaging may improve the early detection of colorectal liver metastases and facilitate dynamic monitoring of treatment response. Among these approaches, targeted molecular ultrasound imaging may offer greater translational potential in CRC, as it enables not only real-time visualization of tumor perfusion but also non-invasive assessment of molecular biomarker expression and therapeutic response. Accordingly, the development of target-specific UTMB platforms represents a promising direction for future CRC diagnosis and precision monitoring. Nevertheless, several limitations still hinder broader clinical application, including insufficient standardization of imaging protocols, limited penetration depth in deep tissues, and the lack of universally validated molecular targets for CRC (Fig. 4).

### Therapy

Overview of the major therapeutic applications of ultrasound-targeted microbubble (UTMB) systems in colorectal cancer (CRC). UTMBs enhance chemotherapy efficacy by increasing vascular permeability, promoting drug accumulation, facilitating sonoporation-mediated intracellular delivery, and reducing systemic toxicity. UTMBs also improve antitumor immunity through immune activation, induction of immunogenic cell death (ICD), and remodeling of the tumor microenvironment (TME), thereby enhancing responses to immune checkpoint blockade. In gene therapy, UTMBs enable targeted delivery of nucleic acids and gene-editing components, including DNA, siRNA, miRNA, and CRISPR systems, leading to modulation of tumor-associated gene expression and tumor suppression. In addition, UTMBs can synergize with other physical treatment modalities, including sonodynamic therapy, photothermal therapy, radiotherapy sensitization, and tumor microvascular disruption. The clinical context of current CRC treatment strategies and their limitations further highlights the therapeutic potential of UTMB-based platforms.

#### Current treatment strategies for colorectal cancer

The management of CRC is stage-dependent. Surgical resection remains the primary treatment for early-stage disease. Locally advanced rectal cancer typically requires neoadjuvant chemoradiotherapy or chemotherapy before surgery. For metastatic CRC, first-line systemic therapy commonly includes FOLFOX or FOLFIRI, often combined with targeted agents such as bevacizumab or cetuximab. ICIs demonstrate clinical efficacy in patients with deficient mismatch repair/microsatellite instability-high (dMMR/MSI-H) tumors; however, most CRC cases are microsatellite stable (MSS) and show limited response. Drug resistance and treatment-related toxicity remain significant challenges, underscoring the need for novel therapeutic platforms such as UTMBs.

#### Chemotherapy intensification

UTMBs enhance the accumulation and penetration of chemotherapeutic agents within the tumor through mechanical and biological effects, while reducing systemic toxicity, thereby improving therapeutic efficacy [61, 87, 88].

**Fig. 5:**
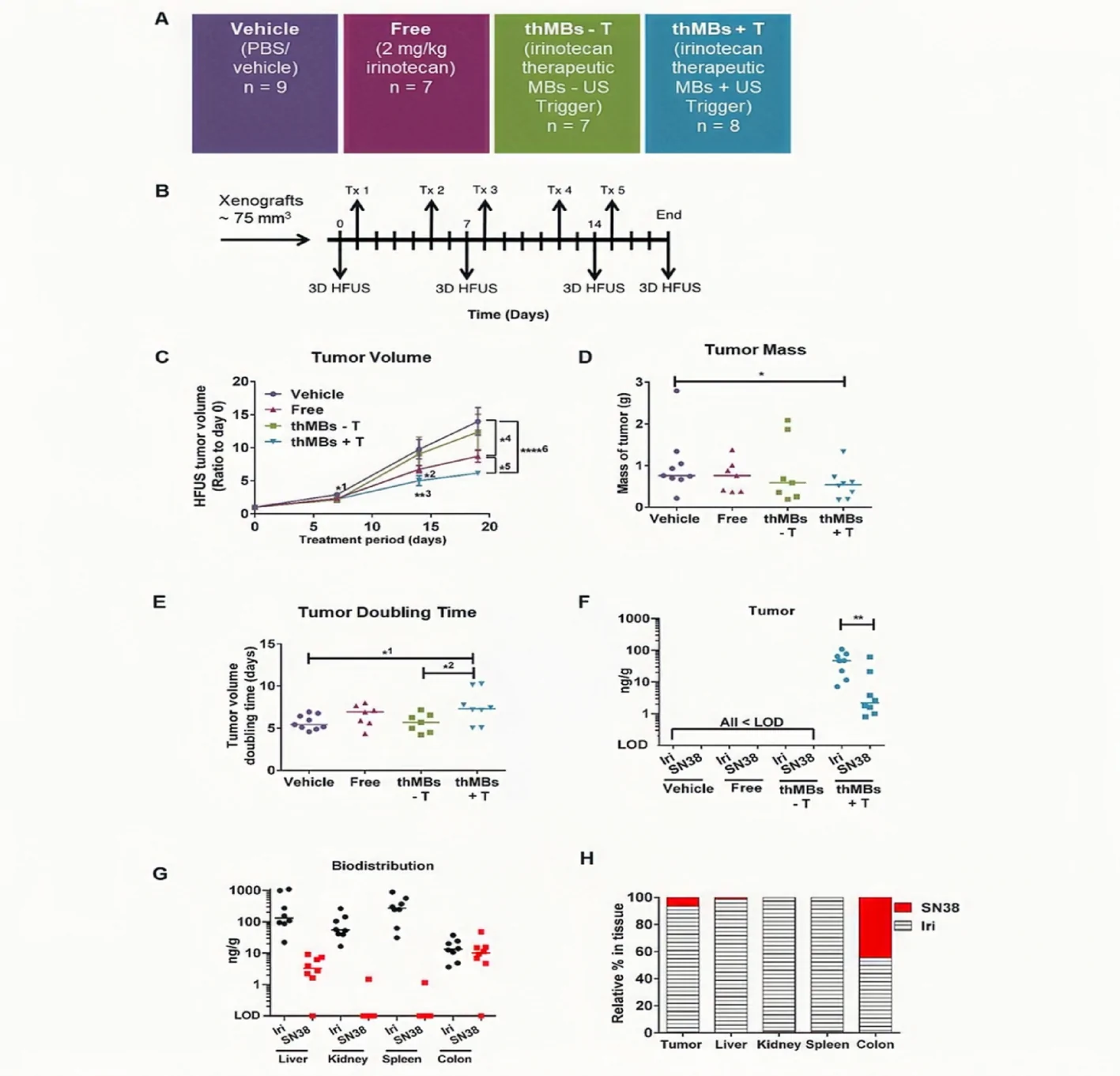
Therapeutic microbubbles (thMBs) enhance the antitumor efficacy of irinotecan in human colorectal cancer xenografts. **A** Tumor volume ratio over time; **B** final tumor mass; **C** tumor drug concentration at 72 h post-treatment. The thMBs + ultrasound-triggered (thMBs+T) group showed significantly reduced tumor growth and increased intratumoral accumulation of irinotecan and its active metabolite SN38 compared with free drug or thMBs without ultrasound treatment. Reprinted from [58] with permission

Enhancing tumor drug accumulation and reducing systemic toxicity: Ultrasound–microbubble interactions increase tumor vascular permeability, promoting drug extravasation into the tumor stroma. Ingram et al. [58] demonstrated that ultrasound-triggered VEGFR2-targeted therapeutic microbubbles (thMBs) increased tumor drug accumulation by up to 2.5-fold while reducing drug distribution in normal tissues by up to 70%, significantly improving the therapeutic index of chemotherapy drugs and limiting their toxicity in healthy tissues. Similarly, Bush et al. [89] reported that Acoustic Cluster Therapy (ACT) combined with irinotecan enhanced drug efficacy by 50% in a murine CRC model through improved drug extravasation, distribution, and cellular uptake. Overcoming Multidrug Resistance (MDR): UTMBs facilitate intracellular drug delivery via sonoporation, thereby bypassing efflux pumps and mitigating MDR. Chen et al. [38] developed porphyrin/ camptothecin-fluorouracil microbubbles (PCF-MBs) that convert into nanoparticles upon ultrasound activation. Significantly enhancing intratumoral drug accumulation and reducing ABCG2 expression, achieving ~90% tumor inhibition in the HT-29 model. Multidrug Combination Delivery: UTMBs enable co-delivery of multiple chemotherapeutic agents, achieving synergistic effects. Gao et al. [37] developed FIRINOX microbubbles carrying 5-fluorouracil, irinotecan, and oxaliplatin. UTMD-mediated FIRINOX delivery significantly delayed tumor growth and improved survival in preclinical models, despite substantially lower drug doses than those used in conventional regimens (Fig. 5).

Clinical trial progress. ACT utilizes microbubble-microdroplet clusters to enhance chemotherapy efficacy. Banerji et al. [90] reported the first phase 1 clinical trial of PS101-based ACT combined with chemotherapy (FOLFOX or FOLFIRI) in patients with CRC liver metastases. The treatment was well tolerated, and ultrasound-treated lesions exhibited greater shrinkage than untreated lesions. Haram et al. [91] further demonstrated that focused ultrasound combined with microbubbles and chemotherapy is safe and feasible, although larger, standardized trials are required. This is consistent with prior evidence summarized by Escoffre et al. [61], who reported that microbubble-enhanced ultrasound demonstrates significant efficacy in preclinical gastrointestinal tumor models and shows promising translational potential in clinical studies of pancreatic ductal adenocarcinoma and liver metastases. Taken together, chemotherapy-assisted delivery represents one of the most mature and clinically translatable therapeutic applications of UTMBs in CRC. By enhancing intratumoural drug accumulation while reducing systemic toxicity, UTMB-based chemotherapy platforms offer a promising strategy for improving the efficacy of conventional CRC treatment.

#### Immunotherapy synergy

UTMBs enhance antitumor immunity by modulating the TME, thereby synergizing with ICI [62, 63]. Importantly, these UTMB-mediated immunomodulatory strategies may be particularly relevant for microsatellite-stable (MSS) CRC, which generally shows limited responsiveness to immune checkpoint blockade alone.

Enhancing immune cell infiltration and activation: UTMD increases the infiltration of CTLs, dendritic cells (DCs), and M1 macrophages, while reducing immunosuppressive populations, including Tregs and MDSCs, thereby reversing the tumor's immunosuppressive microenvironment [59, 60, 64–68]. Jahangiri et al. [64] showed that ultrasound-triggered microbubble cavitation (UTMC) combined with CD39 knockout increased extracellular ATP; this combinatorial strategy synergized with anti-PD-L1 therapy, leading to significant tumor growth inhibition and enhanced infiltration of CTLs, DCs, and M1 macrophages. Li et al. [65] demonstrated that ultrasound-stimulated microbubble cavitation (USMC) combined with PD-L1 blockade significantly inhibited tumor growth and prolonged survival in the MC38 colorectal cancer model, primarily by enhancing tumor perfusion and CD8+ T cell infiltration. Induction of Immunogenic Cell Death (ICD): UTMD can induce ICD, leading to the release of DAMPs, dendritic cell activation, and subsequent antitumor immune responses [67, 69, 70]. Zhao et al. [70] further demonstrated that an acid-responsive nanoscale ultrasound contrast agent triggered nitric oxide (NO) release via UTMD, thereby synergistically enhancing immunotherapy efficacy in combination with paclitaxel-induced ICD. Remodeling the immunosuppressive microenvironment: UTMBs promote M2-to-M1 macrophage polarization, alleviate tumor hypoxia, and modulate the activity of other immunosuppressive cell populations, thereby enhancing antitumor immunity [59, 60, 64, 66, 68, 71, 72]. Zheng et al. [66] demonstrated that a drug-loaded microbubble delivery system (RD@MBs) combined with αPD-L1 effectively remodeled the TME by promoting macrophage polarization, reducing MDSCs, and enhancing immune activation, ultimately inhibiting both primary and metastatic tumor growth. Chen et al. [72] developed ICG@C3F8-R848 nanobubbles for acoustic immunotherapy. UTND enhanced intratumoral drug delivery, while the immune adjuvant R848 induced M2-to-M1 macrophage polarization, thereby amplifying antitumor immunity responses.

Immunomodulation of T cells. Baez et al. [92] demonstrated that microbubble-mediated focused ultrasound modulates T-cell cytokine secretion profiles, including interleukin-1β (IL-1β) and tumor necrosis factor-α (TNF-α), thereby enhancing immune activation and vascular permeability. Gallus et al. [93] showed that combining T-cell therapy with low-intensity pulsed focused ultrasound, microbubbles, and poly-ICLC enhances antigen presentation, disrupts immune tolerance within the central nervous system, and promotes robust T-cell–mediated immune responses. UTMB-assisted immunotherapy represents a promising strategy for overcoming the immunosuppressive tumor microenvironment in CRC. By enhancing immune cell infiltration, promoting immunogenic cell death, and improving the efficacy of immune checkpoint blockade, UTMB-based approaches may help expand the population of CRC patients who can benefit from immunotherapy. This potential may be particularly meaningful for MSS CRC, where effective immunotherapeutic options remain limited and novel combination strategies are urgently needed. However, further studies are still needed to clarify the long-term safety, treatment reproducibility, and optimal combination strategies for clinical translation.

#### Gene therapy

UTMBs enable efficient and targeted delivery of gene therapy vectors to tumor cells and components of the TME through sonoporation-induced transient membrane permeabilization, thereby facilitating gene expression modulation and providing a promising strategy for CRC treatment [45, 94].

Targeted Delivery of Therapeutic Genes: UTMD enables delivery of diverse gene vectors, including plasmid DNA, small interfering RNA (siRNA), microRNA (miRNA), and CRISPR-Cas9 components [95]. Kopechek et al. [44] demonstrated that UTMD-mediated delivery of STAT3 decoy oligonucleotides significantly inhibited STAT3 signaling and tumor growth in squamous cell carcinoma models, primarily by increasing intratumoral gene concentration and enhancing cellular uptake.

Regulation of miRNA expression: miRNAs play a critical role in CRC progression [96]. UTMD facilitates targeted delivery of miRNA mimics or inhibitors, thereby regulating tumor cell proliferation, apoptosis, and invasion. Li et al. [97] demonstrated that UTMD-mediated downregulation of miR-545-3p suppressed CRC cell proliferation and invasion by targeting HOXA3, while promoting apoptosis. Wang et al. [98] reported that UTMD-mediated inhibition of miR-503-5p suppressed CRC progression in vitro through upregulation of SALL1 expression. Li et al. [99] further demonstrated that UTMD enabled efficient delivery of miR-34a mimics, resulting in significant tumor growth inhibition. Regulate gene expression and overcome drug resistance: UTMD can deliver short hairpin RNA (shRNA) or gene overexpression vectors to modulate specific signaling pathways involved in tumor progression and drug resistance. Wang et al. [100] demonstrated that UTMD-mediated overexpression of CNN1 induced ferroptosis in CRC cells via activation of the p53-SLC7A11 pathway, suggesting its potential as both a therapeutic and diagnostic target. Zhang et al. [101] utilized UTMD with siRNA-loaded nanobubbles to silence PDLIM5, thereby revealing its role in autophagy-mediated resistance to gefitinib. Liao et al. [102] demonstrated that UTMD-mediated HIF-1α shRNA delivery combined with transarterial embolization (TAE), effectively suppressed HIF-1α and VEGF expression, resulting in inhibition of tumor growth. Gene editing: Anderson et al. [103] developed a new method that uses focused ultrasound blasting microbubbles (FUTMD) approach for delivering CRISPR-Cas9 base editors to the mouse liver, demonstrating efficient and localized gene editing. Bellary et al. [85] showed that UTMD-mediated gene therapy increased inducible nitric oxide synthase (iNOS) expression, promoting tumor perfusion and enhancing the delivery and efficacy of liposomal doxorubicin in neuroblastoma models, ultimately prolonging survival. Gene therapy is a versatile application of UTMBs in CRC. Among current approaches, miRNA delivery is of particular interest because of its well-established biological basis, broad regulatory functions, and compatibility with ultrasound-triggered targeted delivery. Further studies are needed to improve delivery efficiency, long-term stability, and safety before clinical translation.

#### Other therapeutic applications

Beyond the primary applications mentioned above, UTMBs also synergize with other therapeutic modalities to enhance antitumor effects.

Sonodynamic Therapy (SDT): SDT utilizes ultrasound to activate photosensitizers, generating cytotoxic reactive oxygen species (ROS). UTMBs enhance SDT efficacy by amplifying cavitation effects, improving intracellular drug delivery, and increasing ROS generation [83, 104–107].McKaig et al. [104] demonstrated that UTMD-mediated delivery of docetaxel combined with photosensitizer-based therapy enhanced antitumor efficacy in preclinical models. Chen et al. [107] developed a polymer-wrapped core–shell iron nanoparticle system (PVIBs) loaded with vincristine, integrating chemotherapy, SDT, and Fenton reactions. This system significantly increased ROS production and induced apoptosis in CRC via mitochondria-targeted ultrasound activation.

Photothermal therapy (PTT): PTT employs photothermal agents to generate localized heat under laser irradiation. UTMBs improve PTT efficacy by enhancing targeted accumulation and ultrasound-triggered release of photothermal agents within tumors. Chen et al. [38] demonstrated synergistic photothermal therapy using PCF-MBs under combined ultrasound and laser irradiation. Zhou et al. [108] developed a switchable ROS nano-generator/scavenger system that enhances photothermal efficacy while reducing inflammation through UTMD-mediated delivery.

Radiation therapy sensitization: UTMBs enhance radiotherapy by inducing vascular disruption, improving tumor oxygenation, and increasing radiosensitivity. El Kaffas et al. [109] demonstrated that combining Dll4-Notch pathway inhibition with UTMBs and radiotherapy resulted in synergistic tumor growth delay. Sharma et al. [110] showed that ultrasound-stimulated microbubbles (USMB) combined with low-dose fractionated radiotherapy significantly inhibited tumor growth in human PC3 prostate cancer xenograft models, accompanied by pronounced vascular destruction. Moore-Palhares et al. [111] conducted a phase 1 clinical trial evaluating the safety of MR-guided ultrasound combined with microbubbles (FUS-MB) for radiosensitization in breast cancer, demonstrating a favorable safety profile and potential to improve tumor response and local control.

Tumor microvascular destruction: High-intensity ultrasound combined with microbubbles can directly disrupt tumor microvessels through inertial cavitation, leading to localized ischemia and necrosis and inhibiting tumor growth [41, 50, 94, 109].

Thus, UTMB exhibits broad therapeutic potential in CRC. By integrating with chemotherapy, immunotherapy, gene therapy, and physical treatment modalities, they enhance drug delivery, modulate the tumor microenvironment, and overcome therapeutic resistance, thereby representing a promising platform for precision oncology (Table 2).

**Table 2:** Comparison of UTMB-mediated treatment strategies for CRC

Treatment methods	Mechanism of action of UTMB	Key advantages	Current challenges	Representative literature
Chemotherapy intensification	Increase vascular permeability, acoustic pore effect, and overcome multidrug resistance	Increase tumor drug concentration, reduce systemic toxicity, and enhance therapeutic efficacy	Ultrasound parameter optimization, drug encapsulation efficiency, and clinical translation	[38, 58, 90]
Immunotherapy Synergy	Promote immune cell infiltration, induce ICD, and remodel the TME	Reversing immunosuppression, enhancing ICI efficacy, and inducing systemic immunity	Individual variation in immune response, long-term immune memory, safety	[64–66]
Gene therapy	Sound Hole Effect-Mediated Gene Vector Delivery	High-efficiency targeted gene transfection for gene regulation/editing	Gene delivery efficiency, off-target effects, immunogenicity, safety	[44, 100, 103]

## Challenges and prospects

UTMBs have demonstrated substantial potential for diagnosing and treating CRC. However, several critical challenges remain, necessitating further investigation and technological refinement.

### Challenges

Despite encouraging progress, multiple limitations remain. One of the primary limitations lies in the stability and pharmacokinetic behavior of microbubbles. Conventional micron-sized microbubbles are rapidly cleared by the reticuloendothelial system, and their gas cores are prone to dissolution, resulting in short circulation times in the blood [112]. Although nanobubbles and nanodroplets exhibit prolonged circulation, their structural stability remains to be optimized [21, 113]. In addition, microbubble binding patterns significantly influence biodistribution and in vivo persistence [114]. Strategies to extend circulation time while maintaining targeting stability and avoiding nonspecific aggregation or premature rupture remain to be established. In parallel, targeting specificity and delivery efficiency are significantly affected by tumor heterogeneity, differences in target expression, and the complexity of the TME [14]. Variability in receptor expression across patients, and even within different regions of the same tumor, limits the effectiveness of ligand-based targeting strategies. Consequently, the development of ligands with higher affinity, improved stability, and enhanced tumor penetration is essential. At the same time, low-level expression of target molecules in normal tissues may lead to off-target effects and potential toxicity. Another major challenge involves the optimization and standardization of ultrasound parameters. Parameters such as frequency, intensity, pulse duration, and mechanical index collectively determine microbubble behavior and biological outcomes. Different parameter combinations may either promote stable oscillation or induce inertial cavitation, thereby influencing drug delivery efficiency and safety profiles [18, 61, 105]. The absence of standardized protocols complicates cross-study comparisons and hinders clinical translation. Wu et al. [52, 53] emphasized the need for refined monitoring systems that correlate microbubble dynamics with biological effects, while Frizado and O’Reilly [115] noted the technical challenges of monitoring such processes in complex in vivo environments. Physical limitations, including limited penetration depth and ultrasound attenuation, further restrict UTMB applications. Signal attenuation in deep tissues reduces therapeutic efficiency, particularly for primary CRC lesions or deep-seated metastases [116]. In addition, high-density tissue, such as bone, may interfere with ultrasound propagation and microbubble detection. From a safety perspective, excessive microbubble activity under high-intensity ultrasound may induce tissue damage, hemorrhage, or inflammation [8, 11, 55, 74]. Memari et al. [55] demonstrated that the shear stress generated by microbubble-mediated focused ultrasound can modulate endothelial immune response; however, its potential pro-inflammatory effects require careful evaluation.

Translational challenges remain substantial. The transition from preclinical studies to clinical application involves issues related to large-scale production, quality control, regulatory approval, and cost-effectiveness [21, 61]. Current clinical trials of UTMBs in CRC are still in the early stages, and additional clinical data are needed to establish their safety and efficacy. Furthermore, the intrinsic heterogeneity of CRC represents a major obstacle, as tumors differ significantly among patients and across anatomical sites within the same patient [3]. This heterogeneity limits the universal applicability of a single UTMB-based strategy. Moreover, features of the TME, including immunosuppressive cell populations, hypoxia, and abnormal ECM, may reduce microbubble delivery efficiency and compromise therapeutic outcomes.

### Prospects

Despite these challenges, the future development of UTMBs remains promising. Advances in microbubble design and multi-functionality are expected to improve targeting capability and therapeutic performance. Second, artificial intelligence and machine learning will play a key role in the development and clinical application of UTMB [5, 117, 118]. These approaches can assist in analyzing ultrasound images to improve tumor detection and extract molecular information [5, 116], in optimizing treatment parameters based on patient-specific characteristics to enable precise therapeutic planning, and in facilitating the intelligent screening of novel microbubble materials and targeting ligands. At the same time, personalized precision medicine offers opportunities to tailor UTMB formulations and ultrasound protocols based on genomic, proteomic, and radiomic characteristics, while enabling dynamic monitoring and real-time adjustment of therapeutic strategies. Increasing attention is also being directed toward combination therapies [2, 6, 7, 88], including integration with emerging immunotherapies (e.g., CAR-T cells and oncolytic viruses) to overcome the immunosuppression barrier [93, 119]. As well as the incorporation of gene-editing technologies such as CRISPR/Cas9 for precise genetic modulation [103]. In addition, combination or sequential use with chemotherapy, radiotherapy, and immunotherapy may further enhance efficacy and reduce drug resistance.

Successful clinical translation will require multidisciplinary collaboration, expansion of clinical trials [61, 86, 90, 91, 111], and the establishment of standardized manufacturing, quality control, and regulatory frameworks. Meanwhile, fundamental mechanisms underlying microbubble cavitation effects, ultrasound parameter regulation, and their impact on the TME require further investigation [8, 54, 120, 121]. Such efforts will provide a more robust theoretical foundation for UTMB design and therapeutic strategies.

## Conclusions

UTMBs have demonstrated significant potential as an integrated platform for the diagnosis and treatment of CRC. This review summarizes recent advances in UTMBs, including their diverse formulations, underlying mechanisms, and broad applications in both imaging and therapy.

In the diagnostic setting, UTMBs enhance the detection of primary and metastatic CRC lesions with CEUS, particularly for early identification of liver metastases [15, 75]. Furthermore, molecularly targeted ultrasound imaging enables non-invasive visualization of key biomarkers, such as VEGFR2 and immune checkpoints, including PD-L1 and B7-H3. These capabilities provide valuable tools for early diagnosis, molecular classification, and prediction of therapeutic response to CRC [5, 36].

In therapeutic applications, UTMBs improve the efficacy of multiple anticancer therapies through both physical mechanisms, such as cavitation and acoustic streaming, and modulation of the TME. They enhance the accumulation and penetration of chemotherapeutic agents within tumors, overcome multidrug resistance, and reduce systemic toxicity [37, 38, 58]. In addition, UTMBs exhibit significant potential in immunotherapy by promoting immune cell infiltration, inducing immunogenic cell death, and remodeling the immunosuppressive TME, thereby enhancing the efficacy of ICIs [59, 64, 66]. In the context of gene therapy, UTMBs enable targeted delivery of gene vectors, including miRNA, shRNA, and CRISPR-Cas9 components, via the sonoporation effect, providing novel strategies for gene regulation and editing in CRC [44, 97, 100, 103].

Despite these advances, several challenges remain in the clinical translation of UTMBs. Key issues include microbubble stability, targeting accuracy, optimization and standardization of ultrasound parameters, limited penetration in deep tissues, and potential safety concerns. Moreover, tumor heterogeneity and the complexity of the TME further complicate therapeutic application and limit treatment consistency. Among current applications, targeted molecular imaging and chemotherapy-assisted drug delivery are the most clinically advanced UTMB-based strategies in CRC, supported by relatively mature technical development and increasing clinical evidence. By comparison, immunotherapy- and gene therapy-based approaches are less mature and require further investigation to optimize delivery efficiency, safety, and long-term therapeutic benefit.

Future research is expected to focus on the development of intelligent, multifunctional, and personalized UTMB systems. The integration of artificial intelligence and machine learning into imaging analysis, treatment planning, and drug design will further accelerate progress in this field. In addition, continued exploration of synergistic strategies combining UTMBs with immunotherapy, gene editing technologies, and multimodal treatment approaches will be essential to overcome current limitations. The advancement of large-scale, high-quality clinical trials will also play a critical role in facilitating clinical translation. With continued technological innovation and rigorous validation, UTMBs are expected to become an essential tool in precision oncology, ultimately improving prognosis and quality of life for patients with CRC.

## Data Availability

No datasets were generated or analysed during the current study.
